# Circulating unmethylated *CHTOP* and *INS* DNA fragments provide evidence of possible islet cell death in youth with obesity and diabetes

**DOI:** 10.1186/s13148-020-00906-5

**Published:** 2020-07-31

**Authors:** Farooq Syed, Sarah A. Tersey, Jean-Valery Turatsinze, Jamie L. Felton, Nicole Jiyun Kang, Jennifer B. Nelson, Emily K. Sims, Mathieu Defrance, Martin Bizet, Francois Fuks, Miriam Cnop, Marco Bugliani, Piero Marchetti, Anette-Gabriele Ziegler, Ezio Bonifacio, Bobbie-Jo Webb-Robertson, Appakalai N. Balamurugan, Carmella Evans-Molina, Decio L. Eizirik, Kieren J. Mather, Silva Arslanian, Raghavendra G. Mirmira

**Affiliations:** 1 Center for Diabetes and Metabolic Diseases, Indiana University School of Medicine, Indianapolis, IN USA; 2 Department of Pediatrics, Indiana University School of Medicine, Indianapolis, IN USA; 3 Department of Medicine, Indiana University School of Medicine, Indianapolis, IN USA; 4 Kovler Diabetes Center and Department of Medicine, The University of Chicago, 900 E. 57th Street, KCBD-8130, Chicago, IL 60637 USA; 5 ULB Center for Diabetes Research, Université Libre de Bruxelles, Brussels, Belgium; 6 Laboratory for Cancer Epigenetics, Faculty of Medicine, and ULB Cancer Research Center, Université Libre de Bruxelles, Brussels, Belgium; 7 Division of Endocrinology (ULB Erasmus Hospital), Université Libre de Bruxelles, Brussels, Belgium; 8 Department of Clinical and Experimental Medicine, University of Pisa, Pisa, Italy; 9 Technische Universität München, Munich, Germany; 10 Technische Universität Dresden, Dresden, Germany; 11 Pacific Northwest National Laboratory, Richland, WA USA; 12 Department of Surgery, Cardiovascular Innovation Institute, University of Louisville, Louisville, KY USA; 13 Division of Pediatric General and Thoracic Surgery, Cincinnati Children’s Hospital Medical Center, Department of Surgery, University of Cincinnati, Cincinnati, OH 45229 USA; 14 Roudebush VA Medical Center, Indianapolis, IN USA; 15 Indiana Biosciences Research Institute, Indianapolis, IN USA; 16 Children’s Hospital of Pittsburgh, University of Pittsburgh Medical Center, Pittsburgh, PA USA

**Keywords:** Diabetes, Islet, Biomarker, Cell-free DNA

## Abstract

**Background:**

Identification of islet β cell death prior to the onset of type 1 diabetes (T1D) or type 2 diabetes (T2D) might allow for interventions to protect β cells and reduce diabetes risk. Circulating unmethylated DNA fragments arising from the human *INS* gene have been proposed as biomarkers of β cell death, but this gene alone may not be sufficiently specific to report β cell death.

**Results:**

To identify new candidate genes whose CpG sites may show greater specificity for β cells, we performed unbiased DNA methylation analysis using the Infinium HumanMethylation 450 array on 64 human islet preparations and 27 non-islet human tissues. For verification of array results, bisulfite DNA sequencing of human β cells and 11 non-β cell tissues was performed on 5 of the top 10 CpG sites that were found to be differentially methylated. We identified the *CHTOP* gene as a candidate whose CpGs show a greater frequency of unmethylation in human islets. A digital PCR strategy was used to determine the methylation pattern of *CHTOP* and *INS* CpG sites in primary human tissues. Although both *INS* and *CHTOP* contained unmethylated CpG sites in non-islet tissues, they occurred in a non-overlapping pattern. Based on Naïve Bayes classifier analysis, the two genes together report 100% specificity for islet damage. Digital PCR was then performed on cell-free DNA from serum from human subjects. Compared to healthy controls (*N* = 10), differentially methylated *CHTOP* and *INS* levels were higher in youth with new onset T1D (*N* = 43) and, unexpectedly, in healthy autoantibody-negative youth who have first-degree relatives with T1D (*N* = 23). When tested in lean (*N* = 32) and obese (*N* = 118) youth, increased levels of unmethylated *INS* and *CHTOP* were observed in obese individuals.

**Conclusion:**

Our data suggest that concurrent measurement of circulating unmethylated *INS* and *CHTOP* has the potential to detect islet death in youth at risk for both T1D and T2D. Our data also support the use of multiple parameters to increase the confidence of detecting islet damage in individuals at risk for developing diabetes.

## Background

Diabetes mellitus is a multifactorial disease that occurs following the dysfunction or death of insulin-producing β cells in the pancreas. Globally, there is an alarming increase in the incidence of type 1 diabetes (T1D) and type 2 diabetes (T2D), and it is estimated that 425 million individuals are afflicted with diabetes worldwide [1]. Traditionally, both major forms of diabetes have been viewed as distinct: T1D develops as a result of selective destruction of pancreatic β cells by the immune system, while T2D develops secondary to insufficient insulin secretion in the context of insulin resistance in peripheral tissues. In both forms, clinical manifestations of disease occur only after substantial loss of functional β cell mass, and application of therapeutic interventions at this time point has at best had very limited success in restoring β cell mass and function. Results from our group and others have demonstrated that activation of stress pathways within β cells occurs during the very early phases of both T1D and T2D, resulting in β cell death and/or dysfunction [2–4]; therefore, the noninvasive or minimally invasive detection of β cell death has the potential to serve as an early biomarker of future clinical disease and for monitoring the impact of novel therapeutic interventions.

Recently, we and others have proposed the measurement of circulating unmethylated DNA encoding preproinsulin (*INS*) as a biomarker for β cell damage [5–8]. Given the increased frequency of unmethylated *INS* CpG sites in β cells, the ratio of unmethylated-to-methylated *INS* DNA released into the circulation upon cell death is considered a reflection of β cell death. However, we recently developed a multiplex PCR-based assay using a more precise droplet digital PCR (dPCR) technique to directly quantitate differentially methylated DNA species, and discovered that subjects with new onset (T1D) display significantly elevated levels of both unmethylated and methylated *INS* DNA compared to controls [6, 9]. Notably, although β cells have been identified as containing predominantly unmethylated *INS* DNA, other cell types also contain varying, but lower, levels of unmethylated *INS* [7]. Therefore, the appearance of circulating unmethylated *INS* does not exclusively report on β cell death, and more rigorous or complementary biomarkers are needed.

In an effort to address the current limitations of differentially methylated *INS* as a biomarker for β cell damage, we hypothesized that other differentially methylated genes would show either greater specificity for β cells or could be used as complementary biomarkers to increase β cell specificity. To test this hypothesis, we utilized an unbiased approach leveraging the Infinium HumanMethylation 450 array to identify new differentially methylated CpG targets in human islets. We identified an intragenic CpG site in the gene encoding chromatin target of PRMT1 (*CHTOP*) that exhibits complementary tissue specificity to *INS* and may be used to increase confidence of detecting islet damage in youth with prediabetes and diabetes.

## Results

### Identification of differentially methylated genes from isolated human islet DNA

**Fig. 1:**
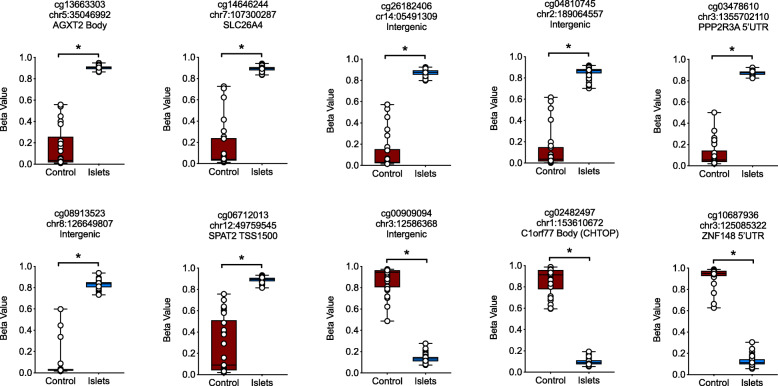
Methylation status of differentially methylated CpG sites identified by Infinium HumanMethylation 450 array. Infinium HumanMethylation 450 array was performed on bisulfite-treated DNA from 64 human islet samples and compared to data from 27 human non-islet tissues (control) obtained from ENCODE DNA methylation datasets (GSE40699). Informatics analysis of these datasets identified 2534 hypomethylated CpG sites and 3667 hypermethylated CpG sites in human islets vs. non-islet tissues (see Suppl. Table S4 for a full list of these CpGs). Shown are the box and whisker plots of the data generated from the top 10 differentially methylated CpG sites. *P < 0.0001 for the comparison shown

**Fig. 2:**
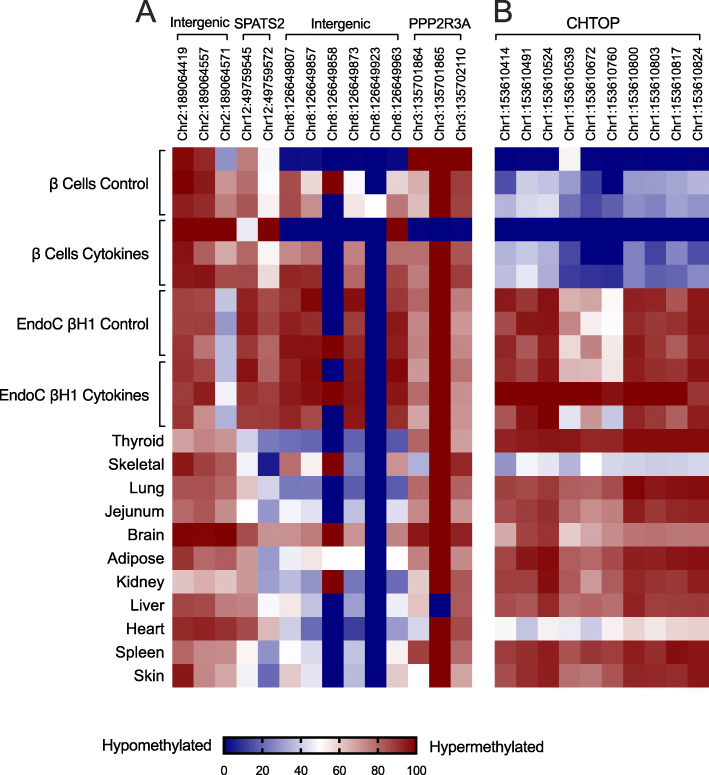
Validation of differentially methylated genes using DNA sequencing. DNA was isolated from Newport Green flow-sorted human β cells (from *N* = 3 independent donors), EndoC-βH1 human fetal β cell-derived line (from *N* = 3 independent cultures), and 11 human control tissues (each from a single donor). DNA sequencing was performed after bisulfite conversion on the top 5 differentially methylated genes identified by the Infinium HumanMethylation 450 array. **a** Heatmap of the relative abundance of methylated (red) and hypomethylated (blue) CpG sites indicated for intergenic, SPATS2, and PPP2R3A genomic locations. **b** Heatmap of the relative abundance of methylated (red) and hypomethylated (blue) CpG sites indicated for the CHTOP gene

To identify genes that exhibit differential methylation in primary human islets, we assessed DNA methylation by Infinium® 450 K array datasets in 64 human islet preparations and leveraged data from 27 publicly available non-islet human tissues (see the “Methods” section). The Infinium® 450 K array covers > 480,000 CpG sites and targets ~ 96% of CpG islands in human genome [10]. Our overall analytic and experimental approach is shown in the flow diagram in Supplemental Fig. S1. Informatics analysis of these datasets identified 2534 hypomethylated CpG sites and 3667 hypermethylated CpG sites in human islets vs. non-islet tissues. The 10 most highly differentially methylated CpG sites are shown in Fig. 1. To verify the methylation status of the identified genes, we performed PCR amplification of a 0.5-kb segment surrounding 5 of the differentially methylated CpG sites (chr12, 49759545; chr2, 189064557; chr8, 126649807; chr3, 1355702110; chr1, 153610672) using bisulfite-treated DNA from fluorescence-sorted primary human β cells (using Newport Green selection, see Supplemental Fig. S2) from 3 different islet preparations, the EndoC-βH1 human β cell-derived line [11] and 11 non-islet human tissues (the remaining 5 differentially methylated CpG sites were not easily amenable to PCR amplification and therefore not pursued further). Products were pooled and deep-sequenced and methylation status of CpG sites was determined. Out of the top 5 differentially methylated CpG sites, CpGs in *CHTOP* (C1orf77) gene, which encodes chromatin target of PRMT1, were found to be substantially differentially methylated in primary sorted human β cells (48–99% hypomethylated) compared to non-islet tissues samples (Fig. 2 a and b). Notable exceptions, however, included heart and skeletal muscle, each of which displayed at least 50% hypomethylation at CpG sites across the *CHTOP* gene. Surprisingly, *CHTOP* CpGs were not hypomethylated in EndoC-βH1 cells (Fig. 2b), consistent with an epigenetic divergence between an embryonic cell line and mature β cells. To determine the stability of CpG site hypomethylation to pro-inflammatory cytokines (inflammatory messengers implicated in diabetes), β cells were treated with IFN-γ and IL-1β for 24 h, and then sequenced. The hypomethylation of CpG sites within the *CHTOP* gene was not altered by cytokine treatment (Fig. 2b).

**Fig. 3:**
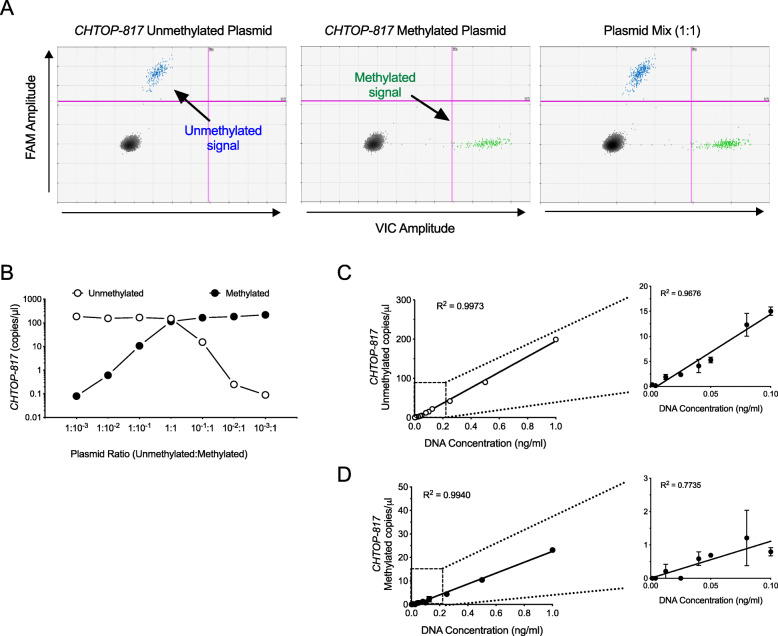
Validation of linearity and sensitivity of dPCR assay for measurement of differential methylation of the CHTOP-817. Plasmid DNA or islet DNA-spiked sera were subjected to droplet dPCR for differentially methylated CHTOP-817 at varying ratios or concentrations. **a** Representative 2-D plots from droplet dPCR output of the gating strategy using the cloned unmethylated and methylated CHTOP gene plasmids. **b** Droplet dPCR quantitation of mixed plasmid dilution of the cloned unmethylated and methylated CHTOP gene plasmids from a representative single experiment (displayed as copies of DNA/microliter). **c** Droplet dPCR quantitation of unmethylated CHTOP-817 after serial dilution of human islet DNA into serum from a healthy human donor (data shown are mean ± SEM from 3 independent dilution experiments from the same islet donor). **d** Droplet dPCR quantitation of methylated CHTOP-817 after serial dilution of human islet DNA into serum from a healthy human donor (data shown are mean ± SEM from 3 independent dilution experiments from the same islet donor). The insets in **c** and **d** show an expanded view of the dilution curve at lower DNA concentrations

Next, we developed TaqMan^TM^ probe-based PCR assays to quantitatively measure differential methylation of the *CHTOP* gene at site chr1, 153610817 (a.k.a. *CHTOP-817*) using droplet digital PCR (dPCR), a technique that allows for absolute quantitation of DNA copy numbers [12]. *CHTOP-817* was selected based on optimal primer and probe characteristics, as determined by the software algorithm at the manufacturer’s website. To determine the sensitivity and linearity of the *CHTOP-817* assay, we first mixed varying proportions of the cloned unmethylated and methylated plasmids of the *CHTOP* gene. Figure 3 a shows the gating strategy in 2-dimensional droplet dPCR plots to detect droplets containing unmethylated and methylated *CHTOP-817*. For verification of linearity and ability to distinguish simultaneous mixtures of the DNA species, mixtures of plasmids at varying ratios were subjected to droplet dPCR as shown in Fig. 3b. To verify linearity of the assay across a range of concentrations seen in circulation, we next spiked various amounts of genomic DNA from human islets into serum from a healthy human donor, as shown in Fig. 3 c and d. Linearity was established for both methylated and unmethylated *CHTOP-817* and preserved below the 2 copies/microliter range (though sensitivity of the methylated *CHTOP-817* assay appeared relatively less linear and less sensitive in this range). Using the linearity validation strategy in Fig. 3 c and d, we created an additional two primer/probe combinations for the *CHTOP* gene to interrogate two other CpG sites (chr1, 153610800 and chr1, 153610824, a.k.a. *CHTOP-800* and *CHTOP-824*, respectively).

**Fig. 4:**
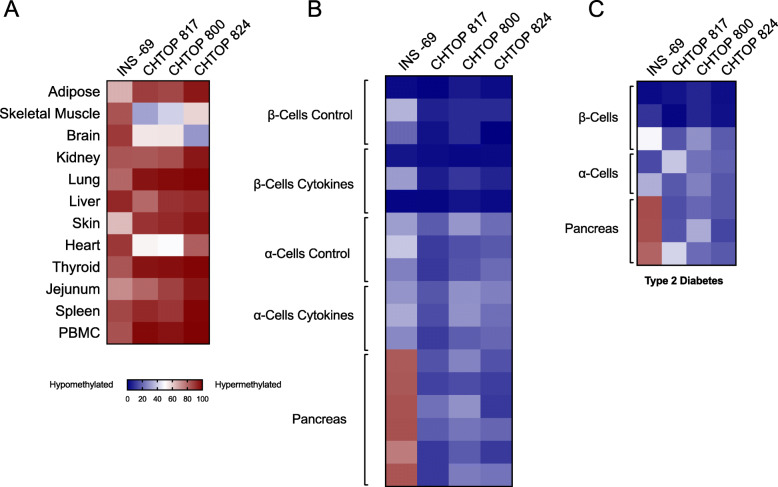
Relative abundance of differentially methylated *CHTOP* and *INS* DNA in human tissue samples by droplet dPCR. DNA from the indicated human tissues was isolated, bisulfite treated, and differentially methylated CHTOP-817, − 800, − 824, and INS DNA levels were quantitated by droplet dPCR. Data are displayed as a heatmap (blue = unmethylated, red = methylated). **a** Non-pancreatic tissues, each from a single donor. **b** Flow-sorted β cells (*N* = 3 donors) treated with and without proinflammatory cytokines (IL-1β and IFN-γ), α cells (*N* = 3 donors) treated with and without proinflammatory cytokines, and total pancreas (*N* = 6 donors). **c** Flow-sorted β cells (*N* = 3 donors) treated with and without proinflammatory cytokines, α cells (*N* = 2 donors) treated with and without proinflammatory cytokines, and total pancreas (N = 3 donors) from subjects with type 2 diabetes

Next, we used each *CHTOP* primer/probe set to test for the relative degree of CpG methylation across a series of non-islet tissues and different pancreatic cell types. For comparison, we also tested the relative degree of CpG methylation at the *INS* gene (CpG at − 69 bp relative to the transcriptional start site) using our previously validated *INS* assay [6]. Figure 4 a shows relative methylation of *INS* and *CHTOP* CpGs in non-pancreatic tissues. Whereas all tissues studied exhibited > 50% methylation at the *INS* site (consistent with the absent *INS* expression in these tissues), it is notable that adipose tissue and skin exhibited only ~ 55–60% methylation—a finding suggesting that excessive damage and/or turnover of these tissues may increase circulating unmethylated *INS* DNA levels. Similarly, for all *CHTOP* sites studied, skeletal muscle, brain, and heart demonstrated ≤ 50% methylation suggesting also that damage and/or turnover of these tissues could increase circulating unmethylated *CHTOP* DNA levels. In pancreatic tissue, *INS* exhibited hypomethylation in both β cells and α cells, but not in whole pancreas (which is largely comprised of acinar cells) (Fig. 4b); by contrast, all *CHTOP* sites exhibited relative hypomethylation in β cells, and to a lesser extent in α cells and whole pancreas. These pancreatic tissue patterns are unaltered by cytokine treatment (Fig. 4b) or in the type 2 diabetic state (Fig. 4c).

Because all *CHTOP* sites studied exhibited similar overall characteristics across the tissues, we restricted the remainder of our analyses to *CHTOP-817*, for which we had the most detailed sensitivity and linearity characteristics (Fig. 3). To evaluate if the utilization of both the *INS* and *CHTOP-817* assays together improves the ability to predict the tissue origin of the circulating DNA, we utilized a Naïve Bayes classifier, which is known to perform well with small sample sizes and fewer features [13]. The Naïve Bayes classifier uses Bayes Theorem to predict the membership of a sample treating each feature as an independent variable, in this case to predict tissue type using single-feature models with *INS* and *CHTOP-817* alone, as well as a two-feature model with both unmethylated *INS* and *CHTOP-817* (see the “Methods” section for details). Four tissue type models were evaluated: (1) β cell, (2) islet (which includes both α and β cells), (3) pancreas (which includes islets and non-islet cells), and (4) β cell vs. α cell. Table 1 shows the average classification accuracy (%) based on 5-fold cross validation repeated 100 times using a Naïve Bayes classifier. In all cases, accuracy is improved when considering both assays together, but 100% accuracy is achieved only for islets (which includes both α and β cells).

**Table 1:** Average classification accuracy based on 100 repetitions of 5-fold cross validation of four tissue-type classifications when using only unmethylated *INS*, only unmethylated *CHTOP-817*, or both unmethylated *INS* + *CHTOP-817* together with a *P* < 10^−30^

	*INS*	*CHTOP-817*	*INS + CHTOP-817*
β cells vs. other tissues	80.6	86	88.8
Islet cells vs. other tissues	98.3	76.8	100
Pancreatic cells vs. other tissues	70.8	92.7	94.8
β cells vs. α cells vs. other tissues	86	68.7	92.7

### Assessment of unmethylated and methylated CHTOP-817 and INS DNA in youth with T1D

**Fig. 5:**
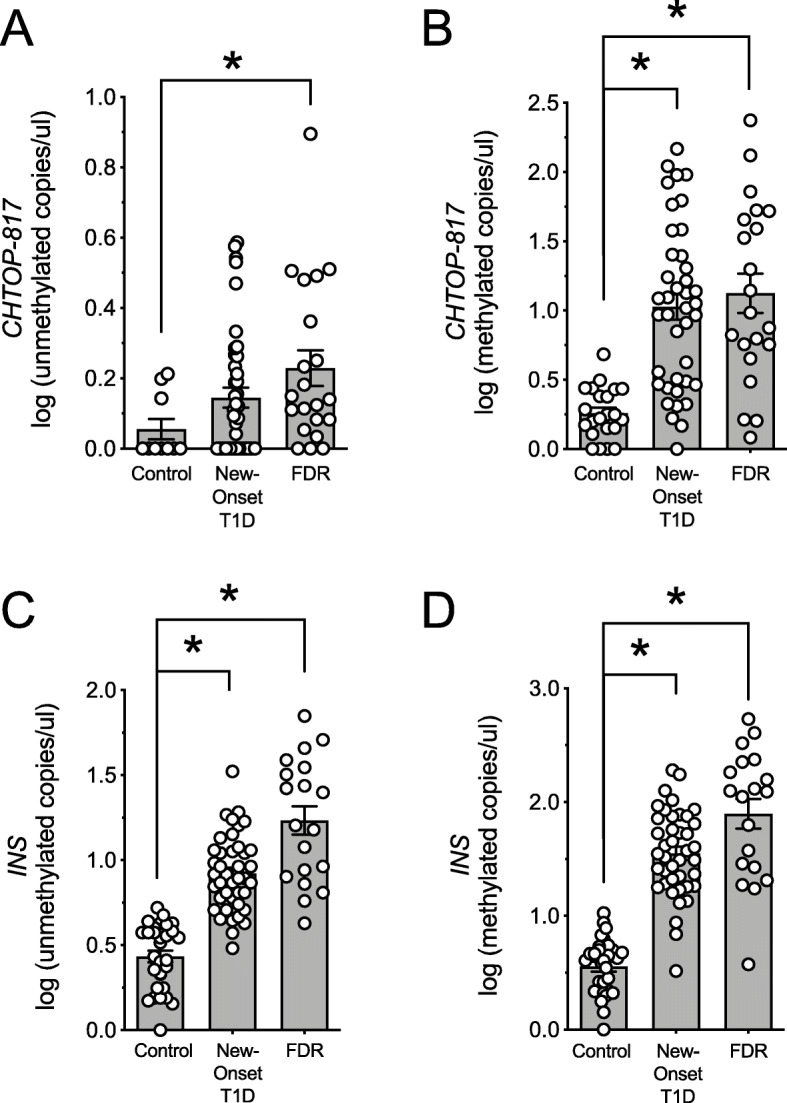
Circulating differentially methylated *CHTOP-817* and *INS* DNA levels in control, new-onset T1D, and autoantibody-negative first-degree relatives (FDRs) of individuals with T1D. DNA was isolated from serum, bisulfite-converted, and differentially methylated *CHTOP-817* and *INS* levels were analyzed by droplet dPCR. **a** Log of unmethylated *CHTOP-817* DNA levels; **b** log of methylated *CHTOP-817* DNA levels; **c** log of unmethylated *INS* DNA levels; **d** log of methylated *INS* DNA levels. Data are presented as mean ± SEM and each symbol represents an individual donor. *P < 0.05 for the comparisons indicated

To validate our assay in human subjects with known islet cell death, we applied both the *CHTOP-817* and *INS* differentially methylated DNA assays to serum from a cohort of subjects with new-onset T1D (within 48 h of diagnosis) and compared them to healthy control subjects. Relevant demographic and laboratory information are presented in Table 2. As shown in Fig. 5a–d, both unmethylated and methylated *CHTOP-817* and *INS* DNA were significantly higher in subjects with new onset T1D compared to healthy controls, a result consistent with our prior studies on *INS* DNA [6]. Remarkably, when tested in first-degree relatives (FDRs) of subjects with T1D who did not have diabetes or evidence of islet autoimmunity (autoantibody negative), both unmethylated and methylated *CHTOP-817* and *INS* were also significantly increased compared to unrelated healthy control subjects (Fig. 5a–d), a result suggesting that underlying genetic risk may give rise to islet cell death.

**Table 2:** Demographic details of controls, youth with T1D, first-degree relatives (FDR), and sepsis cohorts

Youth with T1D and first-degree relatives (FDRs)				
	Control	FDR	New-onset T1D	***P*** value
**Total (% male)**	10 (50)	23 (57)	43 (59)	
**Age (years)**	11 ± 1.1	10 ± 0.4	7.4 ± 0.6	0.126
**Sepsis cohorts**				
	**Control**	**Sepsis**	***P*value**	
**Total (% male)**	10 (60)	10 (60)		
**Age (years)**	14 ± 0.6	11 ± 1.4	0.98

### Assessment of unmethylated and methylated CHTOP-817 and INS DNA in obese youth

**Fig. 6:**
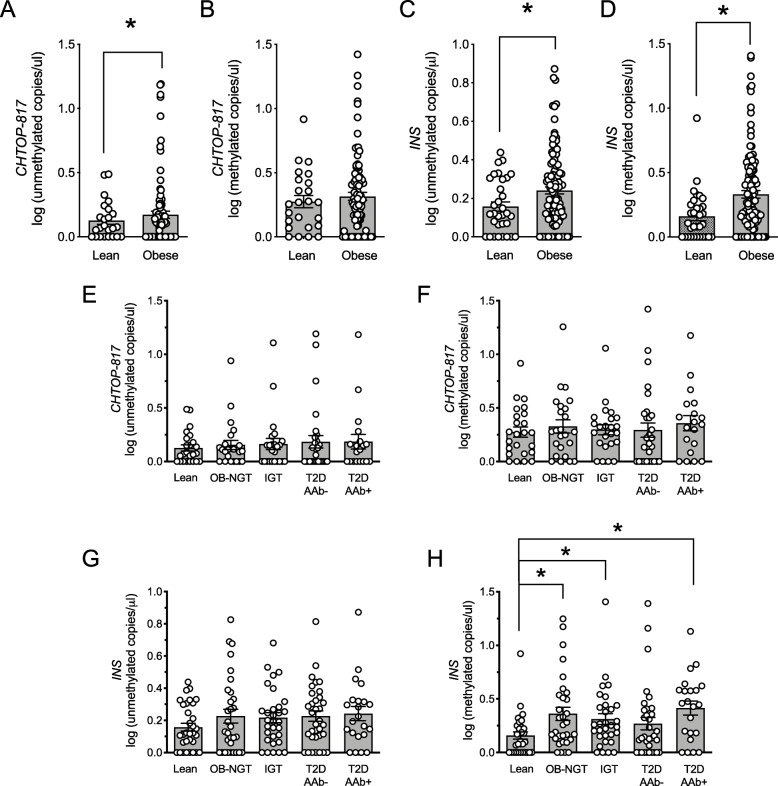
Circulating CHTOP-817 and INS DNA levels in youth with obesity with or without T2D. DNA was isolated from serum, bisulfite-converted, and differentially methylated CHTOP-817 and INS levels were measured by droplet dPCR. **a** Log of circulating unmethylated CHTOP DNA levels in lean and obese youth; **b** log of circulating methylated CHTOP DNA levels in lean and obese youth; **c** log of circulating unmethylated INS DNA levels in lean and obese youth; **d** log of circulating methylated INS DNA levels in lean and obese youth; **e**–**h** log of circulating unmethylated CHTOP-817 (**e**) and INS (**g**) and methylated CHTOP-817 (**f**) and INS (**h**) DNA levels in lean youth with normal glucose tolerance (NGT) and youth with obesity and normal glucose tolerance (OB-NGT), obesity and impaired glucose tolerance (IGT), and clinician-diagnosed obesity with T2D without (T2D-AAb−) and with (T2D-AAb+) autoantibodies. Data are presented as mean ± SEM. *P < 0.05 for the comparisons indicated

To determine if our assays could detect islet cell death associated with insulin resistance, we measured differentially methylated *INS* and *CHTOP-817* DNA levels in cohorts of lean and overweight/obese youth (see clinical characteristics in Table 3). The overweight/obese youth as a group showed significantly higher levels of both unmethylated *CHTOP-817* and *INS*; given that both DNA species show hypomethylation in islet cells, and assuming that the elevated DNA signals are arising from the same cell type, we interpret these data as suggesting the occurrence of islet cell death in the overweight/obese cohort of youth. Methylated *INS* DNA, which arises from non-islet cell types, was also significantly elevated in the overweight/lean cohort compared to lean control youth, but no changes in methylated *CHTOP-817* DNA were observed between groups (Fig. 6a–d).

**Table 3:** Clinical and anthropometric characteristics of youth obesity and T2D cohorts

	Lean	OB-NGT	IGT	AAb− T2D	AAb+ T2D	*P* value*
Total (% male)	32 (56)	31 (35)	31 (35)	34 (47)	22 (45)	
Age, years	13 ± 0.2	14 ± 0.3	15 ± 0.4	15 ± 0.3	14 ± 0.5	< 0.001
BMI, Z score (ZS)	-0.14 ± 0.15	2.21 ± 0.10	2.33 ± 0.06	2.39 ± 0.05	1.90 ± 0.12	< 0.001
HbA1c (%)	5.3 ± 0.1	5.4 ± 0.1	5.4 ± 0.1	6.6 ± 0.1	6.3 ± 0.2	< 0.001
Fasting glucose, mg/dL	95.3 ± 3.5	90.8 ± 3.5	92.5 ± 3.5	115.1 ± 3.4	129.1 ± 5.0	< 0.001
2-h OGTT glucose, mg/dL	N/A	111.4 ± 10.5	158.8 ± 8.4	197.5 ± 8.1	299.1 ± 12.1	< 0.001
Treatment modality *N* (%)						
Lifestyle				7 (21)	2 (13)	
Insulin				4 (12)	3 (19)	
Metformin				16 (47)	2 (13)	
Insulin and metformin				7 (21)	9 (56)	

*P values indicate significance across all cohorts by one-way ANOVA

*OB-NGT* obese normal glucose tolerance, *IGT* impaired glucose tolerance, *AAb−* autoantibody negative, *AAb+* autoantibody positive

To assess if any of these differences were driven by differences in glycemic control, we stratified our cohorts by glycemic control as follows: lean controls with normal glucose tolerance (NGT), overweight/obese with normal glucose tolerance (OB-NGT), overweight/obese with impaired glucose tolerance (IGT), and overweight/obese with T2D with and without evidence of autoantibodies (T2D, AAb+, and T2D-AAb−, respectively). As shown in Fig. 6e–h, there were no statistically significant differences in unmethylated *CHTOP-817* and unmethylated *INS* DNA among these cross-sectional cohorts, suggesting that the overall increases in unmethylated DNA species in the overweight/obese youth reflect a difference largely driven by weight. Methylated *CHTOP-817* DNA was similar across cohorts (Fig. 6f); however, methylated *INS* DNA was significantly elevated in obese youth with normal glucose tolerance (OB-NGT), IGT, and T2D-AAb+ compared to healthy lean controls (Fig. 6h).

### Elevations in methylated INS and CHTOP-817 DNA may be associated with systemic inflammatory states

**Fig. 7:**
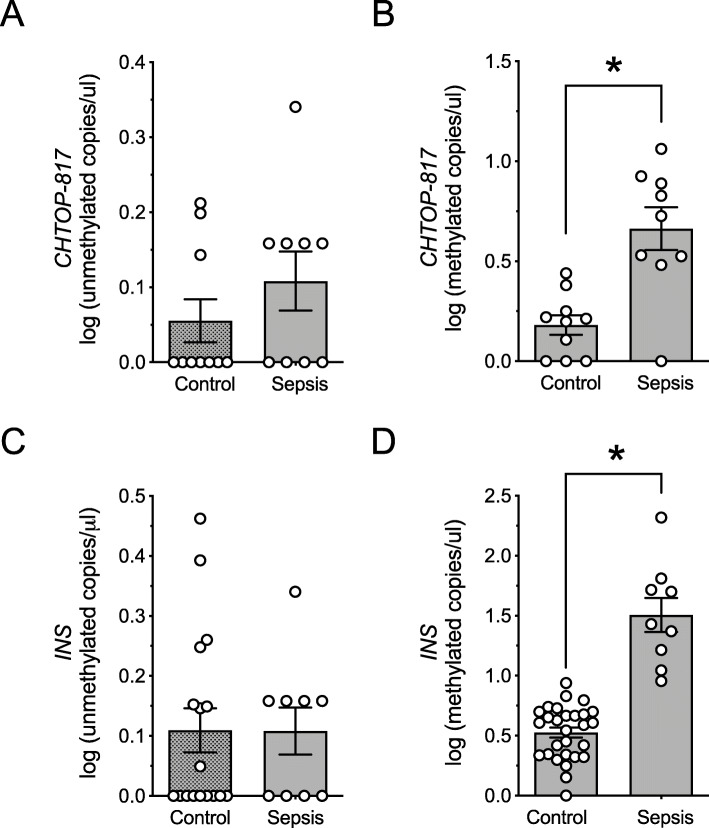
Circulating *CHTOP-817* and *INS* DNA levels in youth with sepsis. DNA was isolated from serum, bisulfite-converted, and differentially methylated *CHTOP-817* and *INS* DNA levels were measured by droplet dPCR. **a** Log of unmethylated *CHTOP-817* DNA levels; **b** log of methylated *CHTOP-817* DNA levels; **b** log of unmethylated *INS* DNA levels; **d** log of methylated *INS* DNA levels. Data are presented as mean ± SEM. *P < 0.05 for the comparisons indicated

The elevation in methylated *INS* in youth with obesity is reminiscent of the elevations in methylated *INS* we reported in youth with new-onset T1D [6]. In this regard, studies in humans suggest that total cell-free DNA levels increase with severity of systemic inflammation/illness in youth [14]. We therefore hypothesized that the elevation in methylated *INS* and *CHTOP-817* in our populations might reflect concurrent systemic inflammation related to the underlying T1D or overweight/obesity. To test this hypothesis, we next applied our assays to sera from youth with severe illness (sepsis requiring intensive care unit-level care) and compared them to age- and sex-matched healthy controls (see Table 2). As shown in Fig. 7a–d, compared to controls, subjects with sepsis exhibited elevations in methylated *INS* and *CHTOP-817* DNA levels (Fig. 7b, d) but not the corresponding unmethylated DNA levels (Fig. 7a, c).

## Discussion

Measurement of circulating differentially-ethylated DNA species has been gaining attention as a minimally invasive biomarker of β cell death that may be used to distinguish individuals with impending and new-onset T1D [6–8, 15–17]. However, owing to the variability of *INS* methylation among various non-β cell types, it cannot be definitively concluded that the signal identified emanates from the islets alone. It is likely that multiple biomarkers will be required to unambiguously detect islet damage. Our study represents a step in this direction. Four key findings emanate from our study: (a) neither differentially methylated *CHTOP* nor *INS* exhibits specificity for the β cell within the islet, as α cells also exhibit nearly the same degree of unmethylation at these (and by inference other) genes, (b) the combination of unmethylated *CHTOP-817* and *INS* DNA levels provides greater confidence that signals are originating from islet cells, (c) autoantibody-negative FDRs of individuals with T1D exhibit a circulating differentially methylated DNA signature similar to individuals with T1D, and (d) youth with overweight/obesity, as a group, exhibit elevated markers consistent with islet cell death and inflammation.

Using an unbiased approach by extracting data from our present and previous studies using the Infinium® 450 K array, we identified *CHTOP* as a gene that was highly differentially methylated in primary human islets. We performed DNA sequencing in non-islet tissues, primary human β cells and α cells, and the validated human β cell line EndoC-βH1. Our finding that both *CHTOP* and *INS* were largely unmethylated in both β cells and α cells is consistent with the common embryonic origin of both cell types, and is further consistent with studies showing that hypomethylation of genes (especially in promoter regions) is a shared feature of α and β cells [18]. We therefore suggest that assays employing differently methylated genes likely report broadly on islet cell death, rather than β cell death specifically. Another important finding in our studies was that *CHTOP* was found to be hypomethylated in primary human β cells, but not in the EndoC-βH1 cells, a human embryo-derived β cell line [11]. This result suggests that while this cell line has proven useful in interrogating β cell biology, its epigenetic landscape may not be reflective of mature β cells.

The interpretation of results of differentially methylated DNA assays most often requires context with regard to the populations being assessed, since they may not be sufficiently specific for β cells to exclude other damaged or dying cell types. In this respect, our results show that other non-islet cell types exhibit at least 50% hypomethylation of the CpG sites in the *CHTOP* and *INS* genes (brain, skeletal muscle, and heart for *CHTOP*, and adipose and skin for *INS*). Others have similarly reported varying degrees of hypomethylation of *INS* across different cell types [7]. In the setting of new/recent-onset T1D and the immediate post-islet transplant period, the suggestion of β cell death is unlikely to be complicated by death of the other cell types studied here, and therefore the interpretation of prior *INS* assays under these conditions has been relatively straightforward [5, 6, 8, 19, 20]. By contrast, under more ambiguous circumstances, such as in individuals at risk for developing T1D or T2D, where the occurrence and timing of β cell death has yet to be defined, these assays may prove more challenging to interpret. For example, we observed that both circulating unmethylated *CHTOP-817* and *INS* were elevated in subjects with new-onset T1D—a finding consistent with the plausibility of active β cell death in these individuals. However, the similar finding of elevated circulating unmethylated *CHTOP-817* and *INS* in autoantibody-negative FDRs of individuals with T1D poses an interpretative challenge. Studies of autoantibody-negative relatives of T1D subjects have shown systemic signatures of prevailing innate immunity [21], and more recently it was demonstrated that autoantibody-negative FDRs of individuals with T1D exhibit reduced pancreatic volume [22]. Moreover, a review of published studies of healthy relatives of individuals with T1D is suggestive of prevailing β cell dysfunction in this group [23]. Thus, our data on FDRs add to the existing literature of abnormalities in these subjects: the combination of elevated circulating unmethylated *CHTOP-817* and unmethylated *INS* in these subjects seemingly excludes death of non-islet cell types (since *CHTOP* and *INS* have non-overlapping patterns of unmethylation outside of the islet, Fig. 4) and raises the specter of islet cell death/turnover in this group.

In T2D, the primary etiology of reduced insulin secretion remains unclear. Autopsy studies have demonstrated increases in β cell mass in adults with obesity and prediabetes compared to controls, and lower β cell mass in individuals with frank T2D compared to controls [24–26]. In obese youth, comprehensive autopsy data are not available, but functional data suggest a worsening of β cell function with increasing dysglycemia [27]. The loss in functional β cell mass in T2D has been attributed variably to β cell apoptosis and/or dedifferentiation [24, 25, 28, 29], but definitive evidence for either is lacking. We discovered that unmethylated *INS* and *CHTOP-817* were significantly elevated in obese youth compared to lean controls, but these increases were lost when the groups were stratified by glycemia (noting that statistical power decreased by the stratification). Given that all individuals are either obese or overweight, it is possible that the unmethylated *INS* signal might be arising from adipocyte turnover; however, the elevations in unmethylated *CHTOP-817*, assuming that they are arising from the same cell type, likely point to an islet cell origin. In a prior study using high-fat diet feeding in mice, we observed that overweight/obese mice exhibit periodic increases in unmethylated *INS* DNA, and that these elevations correlated with increases in β cell death, as verified by immunostaining of pancreas sections from these animals [30]. These data suggest that the elevated unmethylated *INS* and *CHTOP-817* signal in our cohort of overweight/obese youth might be arising from death of islet cells, recognizing that our assay cannot definitively conclude the specific islet cell type giving rise to this signal.

We showed previously that methylated *INS* was elevated in subjects with new-onset T1D; because methylated *INS* (and *CHTOP-817*) have the potential to arise from any cell type, we interpreted these findings as suggestive of turnover of cell types that reflect the general inflammatory state seen in T1D [6]. Here, we demonstrate that youth with severe systemic inflammation/illness (sepsis) exhibit elevated methylated *CHTOP-817* and *INS*, without changes in corresponding unmethylated DNA levels. Our data are consistent with prior reports that suggest that systemic illnesses lead to elevations in circulating cell-free DNA levels [14].

## Conclusions

In closing, some caveats to our study should be noted. First, our studies in humans utilized cross-sectional cohorts, and we cannot comment on the occurrence or the timing of islet cell death following initial islet assault during the progression from obesity to T2D or from antibody-negative FDR to T1D. Given the short (~ 90 min) half-life of DNA in the circulation [15], if β cell death was episodic during disease progression in humans (as we observed previously in mice [30]), signals corresponding to cell death could be missed depending on the timing of sampling. Additional studies measuring differentially methylated *INS* and *CHTOP* from longitudinal cohorts are needed to resolve this issue. Second, neither our unmethylated *CHTOP* nor *INS* assays can reliably distinguish β cell death vs. death of other islet cell types; this caveat may well apply to all cell-free DNA assays developed to date. Thus, it is imperative that all cell-free DNA assays rigorously test their specificity for different human tissues (as we have done here). Taken together, our data suggest that genes not known to be exclusively expressed in islets may still serve as potential biomarkers of islet cell death. The combination of multiple such biomarkers may help to increase specificity of their correlation to β cell/islet cell death and may provide insight into the cellular pathophysiology of diabetes.

## Methods

### Isolation of primary human β cells

Dissociation of human islets was achieved by incubation with accutase (Millipore) supplemented with 5 U/ml of DNAse 1 at 37 °C for 10–15 min. The dissociated cells were washed with 1% BSA in PBS and cultured in islet standard medium (Prodo labs) and followed by Newport Green labeling [31]. β cells and non-β cells were sorted by positive or negative Newport Green staining (respectively) using a BD FACSAria cell sorter (BD Biosciences). The quality of the sorted cells was further confirmed by immunofluorescence staining of insulin (guinea pig anti-insulin, Dako) and glucagon (mouse anti-glucagon, Abcam).

### Methylation-specific DNA sequencing

We performed a methylation specific Infinium HumanMethylation 450 array (Illumina) of 64 human islet preparations (see Suppl. Table S1 for sample details) and compared them with 27 human tissues/cell lines; the control data were retrieved from the Encyclopedia of DNA Elements (ENCODE) [32] DNA methylation database using GEO repository GSE40699 (see Suppl. Table S2 for sample details). The Infinium data (human islets and ENCODE controls) were filtered by removing low-quality data using a detection *P* value threshold of 0.05. Cross-reactive probes (those that co-hybridize at different genomic locations) and probes containing SNPs were filtered out using the extended annotation as previously described [33]. Beta values were computed using the formula Beta value = M/(U + M) where M and U are the raw “methylated” and “unmethylated” signals, respectively.

For the human tissues/cell control data, the first selection of markers was performed by detecting CpG sites showing a significant differential methylation between the two groups (human islets vs non-islet tissues). CpGs with an absolute delta beta > 0.5 (i.e., difference between average methylation levels in each group) and a corrected *P* value < 0.001 computed using Mann–Whitney *U* test followed by Benjamini-Hochberg multi-testing correction were selected. CpGs displaying a delta beta > 0.5 (i.e., methylation difference higher than 50% between the islet group and the control group) were considered as hypermethylated, while the CpGs displaying a delta beta ≤ 0.5 where considered as hypomethylated (see Suppl. Table S4 for a full list of all hyper- and hypomethylated CpGs, their respective delta beta values and *P* values).

For experimental confirmation of differentially methylated CpGs, DNA was isolated from sorted human β cells, EndoC βH1 cells, and human tissues (brain, heart, lung, thyroid, spleen, intestine, skin, skeletal muscle, adipose, pituitary, pancreas, and liver) following manufacturer’s protocol (GenElute™ Mammalian Genomic DNA Miniprep kits, Sigma). Following isolation, DNA was bisulfite treated using EZ DNA Methylation-Lightning Kit (Zymo Research), and PCR was performed using primers specifically designed to amplify bisulfite-converted DNA (Suppl. Table S3). Primers were designed using the MacVector® software (MacVector, Inc.). PCR products were purified using a QIAquick PCR purification kit (Qiagen) and DNA was quantified using a Qubit dsDNA assay kit (Invitrogen). Equal amounts of DNA were used for library preparation. Fully methylated or unmethylated synthetic DNA was used as a positive control to calculate the degree of methylation. Methylation-specific DNA sequencing was performed using an Ion Proton System (LifeTechnologies).

### Plasmid synthesis

Unmethylated and methylated *CHTOP* and *INS*-containing plasmids were constructed from PCR-generated products using a TOPO^TM^ TA Cloning kit (Thermo Fisher) DNA from β cells and non-β cells. The TOPO-DNA constructs were transformed into *E.coli* and cultured overnight on LB plates containing ampicillin. Single colonies were handpicked for miniprep cultures and DNA from bacteria was isolated using an QIAprep Spin Miniprep kit (Qiagen). The plasmid sequence was confirmed by DNA sequencing (Sanger) using M13R primers.

### Methylation specific multiplex droplet dPCR assay

TaqMan® probes were designed for the interrogation of differential methylation pattern at CpG sites chr1, 153610817; chr1, 153610800; and chr1, 153610824 of the *CHTOP* gene by multiplex droplet dPCR. Primers and probes were as follows: chr1, 153610817: forward, 5′-TTTGGAGTTTTTGGTTTAGTAAGTTATGAAAATGTT; reverse, 5′-CATCTACTAAACCAATCTTCTATTTCTAACACTAACTAA; VIC probe, 5′-AAACCCGAATATTCAC; FAM probe, 5′-AAACCCAAATATTCAC. chr1, 153610800: forward, 5′-TTTGGAGTTTTTGGTTTAGTAAGTTATGAAAATGTT; reverse, 5′-CATCTACTAAACCAATCTTCTATTTCTAACACTAACTAA; VIC probe, 5′-AATATGTTGAAGAATAAATAGTCGA; FAM probe, 5′-AATATGTTGAAGAATAAATAGTTGA. chr1, 153610824: forward, 5′-TTTGGAGTTTTTGGTTTAGTAAGTTATGAAAATGTT; reverse, 5′-CCATCTACTAAACCAATCTTCTATTTCTAACACT; VIC probe, 5′-ACTACATCGAAACCC; FAM probe, 5′-ACTACATCAAAACCC. Primers and probes for the *INS* gene (site—69 bp relative to the transcriptional start site) were described previously [6, 9]. Assay linearity was determined by serial dilution of unmethylated and methylated plasmids. For the calculation of relative methylation in heatmaps, the ratio of abundance of the unmethylated to methylated DNA fragments was obtained by dPCR and assigned a color along a gradient (blue = hypomethylated to red = hypermethylated) using the GraphPad Prism 7.0c software (GraphPad).

### Human subjects

The samples in the T1D/first-degree relative (FDR) cohort are from the DiMelli and TeenDiab studies. The DiMelli study included subjects with incident T1D in childhood in Bavaria, Germany [34]. Samples from 43 subjects (mean age 7.4 years) who were positive for one or more islet autoantibodies were selected for the study. Samples from 23 islet autoantibody-negative first-degree relatives (FDRs) (mean age 10 years) of individuals with T1D were obtained from the TeenDiab Study [35]. All samples were collected with informed consent by the patients or their legal parents/guardians. The DiMelli study was approved by the Bayerische Landesaerztekammer and the TeenDiab study by the Ludwig-Maximilians University.

Frozen fasting serum samples from 150 youth, ages 10 to < 20 years old, who participated in NIH-funded K24 grant of “Childhood Insulin Resistance” were used for the obesity/overweight/T2D cohort. Data unrelated to the present project have been published [27, 36–40]. Participants were recruited through newspaper advertisements, flyers posted in the medical campus, city bus routes, and the outpatient clinics in the Weight Management and Wellness Center and the Division of Pediatric Endocrinology at the Children’s Hospital of Pittsburgh. The study was approved by the institutional review board of the University of Pittsburgh and written informed parental consent and child assent were obtained from all participants before any research participation in accordance with the ethical guidelines of Children’s Hospital of Pittsburgh. A 2-h OGTT was performed in obese participants as described before [36]. GAD 65 kDa autoantibody and insulinoma-associated protein 2 autoantibody (IA2) were measured using the NIDDK standardized assay protocol as described before [40]. Participants with diabetes were on either lifestyle only, or metformin or metformin plus insulin (Table 3).

Samples from control youth and youth with sepsis were obtained after informed consent and approved through the Indiana University Institutional Review Board.

### DNA isolation and bisulfite conversion

DNA from tissue and cell samples was isolated using GenElute Mammalian Genomic DNA Miniprep Kit (Sigma-Aldrich). DNA from serum was isolated from 50 μl of serum samples spiked in with 5 μg carrier DNA (poly-A) using QIAamp DNA Blood Mini Kit (Qiagen). DNA recovery from serum samples (of the poly-A carrier) was quantified using a nanophotometer (Implen). All samples showed ≥ 85% recovery of DNA following isolation. DNA bisulfite conversion was carried out using EZ DNA Methylation-Lightning Kit (Zymo Research), and conversion was verified using a pre- and post-conversion sample in the droplet dPCR.

### Statistical analysis

All data are presented as mean ± SEM. For analysis of methylated and unmethylated *INS* DNA levels, a Kruskal-Wallis (non-parametric) test was employed followed by a Dunnett’s posttest (to compare values to healthy controls). GraphPad Prism Version 7.0c (GraphPad Software) was used for statistical analyses of sample data. Statistical significance was assumed at *P* < 0.05.

To evaluate if the utilization of *INS* and *CHTOP* assays together improves the ability to predict the tissue type, we utilized a Naïve Bayes classifier to predict tissue type specificity using single-feature models with *INS* and *CHTOP* alone, as well as a two-feature model with *INS* and *CHTOP*. Five-fold cross validation (CV) was preformed and each sample was classified based on the posterior probability at a threshold of 0.5. Once all samples were classified, the classification accuracy was computed as the proportion correctly classified. Four tissue type specificity models were evaluated: (1) beta cell specific; (2) islet cell specific, which includes alpha and beta cells; (3) pancreas specific, which included islet and non-islet cells; and (4) islet cell-type (β cells vs. α cells) specific. To assure that the results were not biased by the random CV selection, the 5-fold CV process was performed 100 times. The same CV datasets were used for the single and double models. A non-parametric test was utilized to compare the accuracy of the *INS*, *CHTOP*, and BOTH assay evaluation. Thus, the 100 observed classification accuracy estimates were compared across assays using a Friedman test to account for the repeated measures in a non-parametric test. A subsequent Tukey-Cramer post hoc test was used to determine the statistical significance between the three comparisons.

## Supplementary information

**Additional file 1.** Supplemental Table S1: Human Islet Donor Characteristics. Supplemental Table S2: Human tissues/cell lines used as controls for Illumina 450-Array. Supplemental Table S3: Primers for sequencing. Supplemental Table S4: Table of all differentially methylated CpGs. Supplemental Figure S1: Experimental Workflow. Supplemental Figure S2: Immunofluorescence staining of flow-sorted human β cells.

## Data Availability

The data generated from methylation specific Infinium HumanMethylation 450 array (Illumina) of 64 human islet preparations are available at GEO (accession number “GSE143209” with reviewer token “gdglekewvpodnyr”).

## Abbreviations

AAb: Autoantibody
*CHTOP*: Gene encoding chromatin target of PRMT1
dPCR: Digital PCR
IGT: Impaired glucose tolerance
*INS*: Gene encoding preproinsulin
NGT: Normal glucose tolerance
OB-NGT: Overweight/obese and normal glucose tolerance
T1D: Type 1 diabetes
T2D: Type 2 diabetes

## References

[1] International Diabetes Federation (2017). IDF Diabetes Atlas [Internet].

[2] Back SH, Kaufman RJ (2012). Endoplasmic reticulum stress and type 2 diabetes. Annu Rev Biochem..

[3] Marhfour I, Lopez XM, Lefkaditis D, Salmon I, Allagnat F, Richardson SJ (2012). Expression of endoplasmic reticulum stress markers in the islets of patients with type 1 diabetes. Diabetologia..

[4] Sims EK, Chaudhry Z, Watkins R, Syed F, Blum J, Ouyang F (2016). Elevations in the fasting serum proinsulin-to-C-peptide ratio precede the onset of type 1 diabetes. Diabetes Care..

[5] Akirav EM, Lebastchi J, Galvan EM, Henegariu O, Akirav M, Ablamunits V (2011). Detection of β cell death in diabetes using differentially methylated circulating DNA. Proc Natl Acad Sci USA..

[6] Fisher MM, Watkins RA, Blum J, Evans-Molina C, Chalasani N, DiMeglio LA (2015). Elevations in circulating methylated and unmethylated preproinsulin DNA in new-onset type 1 diabetes. Diabetes..

[7] Husseiny MI, Kaye A, Zebadua E, Kandeel F, Ferreri K (2014). Tissue-specific methylation of human insulin gene and PCR assay for monitoring beta cell death. PLoS ONE..

[8] Lehmann-Werman R, Neiman D, Zemmour H, Moss J, Magenheim J, Vaknin-Dembinsky A (2016). Identification of tissue-specific cell death using methylation patterns of circulating DNA. Proc Natl Acad Sci USA..

[9] Tersey SA, Nelson JB, Fisher MM, Mirmira RG (2016). Measurement of differentially methylated INS DNA species in human serum samples as a biomarker of islet β cell death. J Vis Exp.

[10] Bibikova M, Barnes B, Tsan C, Ho V, Klotzle B, Le JM (2011). High density DNA methylation array with single CpG site resolution. Genomics..

[11] Scharfmann R, Pechberty S, Hazhouz Y, von Bülow M, Bricout-Neveu E, Grenier-Godard M (2014). Development of a conditionally immortalized human pancreatic β cell line. J Clin Invest..

[12] Hindson CM, Chevillet JR, Briggs HA, Gallichotte EN, Ruf IK, Hindson BJ (2013). Absolute quantification by droplet digital PCR versus analog real-time PCR. Nat Methods..

[13] Yang F-J. An implementation of Naive Bayes classifier. 2018 Int Conf Comput Sci Comput Intell CSCI. IEEE; 2018. p. 301–306.

[14] Moreira VG, Prieto B, Rodríguez JSM, Álvarez FV (2010). Usefulness of cell-free plasma DNA, procalcitonin and C-reactive protein as markers of infection in febrile patients. Ann Clin Biochem..

[15] Herold KC, Usmani-Brown S, Ghazi T, Lebastchi J, Beam CA, Bellin MD (2015). β cell death and dysfunction during type 1 diabetes development in at-risk individuals. J Clin Invest..

[16] Olsen JA, Kenna LA, Spelios MG, Hessner MJ, Akirav EM (2016). Circulating differentially methylated amylin DNA as a biomarker of β-cell loss in type 1 diabetes. PLOS ONE..

[17] Sklenarova J, Petruzelkova L, Kolouskova S, Lebl J, Sumnik Z, Cinek O (2017). Glucokinase gene may be a more suitable target than the insulin gene for detection of β cell death. Endocrinology..

[18] Neiman D, Moss J, Hecht M, Magenheim J, Piyanzin S, Shapiro AMJ (2017). Islet cells share promoter hypomethylation independently of expression, but exhibit cell-type-specific methylation in enhancers. Proc Natl Acad Sci U S A..

[19] Bellin MD, Clark P, Usmani-Brown S, Dunn TB, Beilman GJ, Chinnakotla S (2017). Unmethylated insulin DNA is elevated after total pancreatectomy with islet autotransplantation: assessment of a novel beta cell marker. Am J Transpl..

[20] Roels S, Costa OR, Tersey SA, Stangé G, De Smet D, Balti EV (2019). Combined analysis of GAD65, miR-375, and unmethylated insulin DNA following islet transplantation in patients with T1D. J Clin Endocrinol Metab..

[21] Chen Y-G, Cabrera SM, Jia S, Kaldunski ML, Kramer J, Cheong S (2014). Molecular signatures differentiate immune states in type 1 diabetic families. Diabetes..

[22] Campbell-Thompson ML, Filipp SL, Grajo JR, Nambam B, Beegle R, Middlebrooks EH (2019). Relative pancreas volume is reduced in first-degree relatives of patients with type 1 diabetes. Diabetes Care..

[23] Sims EK, DiMeglio LA (2019). Cause or effect? A review of clinical data demonstrating beta cell dysfunction prior to the clinical onset of type 1 diabetes. Mol Metab..

[24] Butler AE, Dhawan S, Hoang J, Cory M, Zeng K, Fritsch H, et al. β-cell deficit in obese type 2 diabetes, a minor role of β-cell dedifferentiation and degranulation. J Clin Endocrinol Metab. 2015;jc.2015-3566. 10.1210/jc.2015-3566 PMC4880126 26700560

[25] Butler AE, Janson J, Bonner-Weir S, Ritzel R, Rizza RA, Butler PC. β-cell deficit and increased β-cell apoptosis in humans with type 2 diabetes. Diabetes. 2003;52:102–10. 10.2337/diabetes.52.1.102 12502499

[26] Rahier J, Guiot Y, Goebbels RM, Sempoux C, Henquin JC (2008). Pancreatic β-cell mass in European subjects with type 2 diabetes. Diabetes Obes Metab..

[27] Bacha F, Lee S, Gungor N, Arslanian SA (2010). From pre-diabetes to type 2 diabetes in obese youth. Diabetes Care..

[28] Cinti F, Bouchi R, Kim-Muller JY, Ohmura Y, Sandoval PR, Masini M (2016). Evidence of β-cell dedifferentiation in human type 2 diabetes. J Clin Endocrinol Metab..

[29] White MG, Marshall HL, Rigby R, Huang GC, Amer A, Booth T (2013). Expression of mesenchymal and α-cell phenotypic markers in islet β-cells in recently diagnosed diabetes. Diabetes Care..

[30] Tersey SA, Levasseur EM, Syed F, Farb TB, Orr KS, Nelson JB (2018). Episodic β-cell death and dedifferentiation during diet-induced obesity and dysglycemia in male mice. FASEB J..

[31] Lukowiak B, Vandewalle B, Riachy R, Kerr-Conte J, Gmyr V, Belaich S (2001). Identification and purification of functional human beta-cells by a new specific zinc-fluorescent probe. J Histochem Cytochem..

[32] Davis CA, Hitz BC, Sloan CA, Chan ET, Davidson JM, Gabdank I (2018). The Encyclopedia of DNA elements (ENCODE): data portal update. Nucleic Acids Res..

[33] Price ME, Cotton AM, Lam LL, Farré P, Emberly E, Brown CJ (2013). Additional annotation enhances potential for biologically-relevant analysis of the Illumina Infinium HumanMethylation450 BeadChip array. Epigenetics Chromatin..

[34] Thümer L, Adler K, Bonifacio E, Hofmann F, Keller M, Milz C (2010). German new onset diabetes in the young incident cohort study: DiMelli study design and first-year results. Rev Diabet Stud RDS..

[35] Ziegler A-G, Meier-Stiegen F, Winkler C, Bonifacio E, TEENDIAB Study Group (2012). Prospective evaluation of risk factors for the development of islet autoimmunity and type 1 diabetes during puberty--TEENDIAB: study design. Pediatr Diabetes..

[36] Burns SF, Bacha F, Lee SJ, Tfayli H, Gungor N, Arslanian SA (2011). Declining β-cell function relative to insulin sensitivity with escalating OGTT 2-h glucose concentrations in the nondiabetic through the diabetic range in overweight youth. Diabetes Care..

[37] George L, Bacha F, Lee S, Tfayli H, Andreatta E, Arslanian S (2011). Surrogate estimates of insulin sensitivity in obese youth along the spectrum of glucose tolerance from normal to prediabetes to diabetes. J Clin Endocrinol Metab..

[38] Michaliszyn SF, Mari A, Lee S, Bacha F, Tfayli H, Farchoukh L (2014). β-cell function, incretin effect, and incretin hormones in obese youth along the span of glucose tolerance from normal to prediabetes to type 2 diabetes. Diabetes..

[39] Sjaarda L, Lee S, Tfayli H, Bacha F, Bertolet M, Arslanian S (2013). Measuring β-cell function relative to insulin sensitivity in youth: does the hyperglycemic clamp suffice?. Diabetes Care..

[40] Tfayli H, Bacha F, Gungor N, Arslanian S (2010). Islet cell antibody-positive versus -negative phenotypic type 2 diabetes in youth: does the oral glucose tolerance test distinguish between the two?. Diabetes Care..

