# Supplementary material for: Circulating unmethylated CHTOP and INS DNA fragments provide evidence of possible islet cell death in youth with obesity and diabetes
Source: Clin Epigenetics. 2020 Jul 31;12:116. doi: 10.1186/s13148-020-00906-5 (PMC7393900; doi:10.1186/s13148-020-00906-5)
Supplement: Supplementary file 1 — Additional file 1. Supplemental Table S1: Human Islet Donor Characteristics. Supplemental Table S2: Human tissues/cell lines used as controls for Illumina 450-Array. Supplemental Table S3: Primers for sequencing. Supplemental Table S4: Table of all differentially methylated CpGs. Supplemental Figure S1: Experimental Workflow. Supplemental Figure S2: Immunofluorescence staining of flow-sorted human β cells. [file 13148_2020_906_MOESM1_ESM.zip › Additional file/Supplemental data.docx]

**Supplemental Table S1: Human Islet Donor Characteristics**

| Sample | Identifier | Age | BMI | HbA1c | Glycemia | History of Diabetes | Sex | Diabetes Duration |
| --- | --- | --- | --- | --- | --- | --- | --- | --- |
| Illumina | 6929793122_R02C02 | 74 | 23.1 | N/A | 156 | No | M | N/A |
| Illumina | 6929793122_R05C01 | 87 | 27.7 | N/A | 178 | No | M | N/A |
| Illumina | 6929793122_R06C02 | 22 | 22.9 | N/A | 179.5 | No | M | N/A |
| Illumina | 6929793129_R01C01 | 52 | 25.2 | N/A | N/A | No | M | N/A |
| Illumina | 6929793122_R04C01 | 71 | 25.4 | N/A | 161 | No | M | N/A |
| Illumina | 6929793129_R03C01 | 36 | 26.3 | N/A | N/A | No | M | N/A |
| Illumina | 6929793129_R01C02 | 53 | N/A | N/A | 125 | No | M | N/A |
| Illumina | 6929793122_R02C01 | 58 | 24.5 | N/A | 150.5 | No | M | N/A |
| Illumina | 6929793139_R05C01 | 82 | 25.3 | N/A | 165 | No | M | N/A |
| Illumina | 6929793139_R06C02 | 67 | 24.2 | N/A | 181 | No | M | N/A |
| Illumina | 6929793122_R01C01 | 52 | 24.5 | N/A | 113 | No | M | N/A |
| Illumina | 7471147018_R04C01 | 57 | 24.7 | N/A | 114 | No | M | N/A |
| Illumina | 7471147018_R06C02 | 53 | 23.5 | N/A | 138 | No | M | N/A |
| Illumina | 6929742038_R05C01 | 69 | 21.2 | N/A | 122.5 | No | M | N/A |
| Illumina | 6929742057_R06C02 | 47 | 28.4 | N/A | 134.5 | No | M | N/A |
| Illumina | 6929806060_R03C01 | 63 | 27.8 | N/A | N/A | No | M | N/A |
| Illumina | 7471147046_R05C02 | 58 | 24.5 | N/A | 148 | No | M | N/A |
| Illumina | 6929806060_R04C02 | 75 | 29.4 | N/A | 165 | No | M | N/A |
| Illumina | 6929742038_R06C02 | 61 | 27.8 | N/A | N/A | No | F | N/A |
| Illumina | 6929806060_R01C01 | 67 | 30.4 | N/A | 113 | No | M | N/A |
| Illumina | 6929742057_R04C02 | 71 | 27.7 | N/A | N/A | No | M | N/A |
| Illumina | 7471147042_R01C01 | 56 | 27.8 | N/A | 129 | No | M | N/A |
| Illumina | 7471147042_R02C02 | 58 | 25.6 | N/A | 338 | No | M | N/A |
| Illumina | 6929742038_R06C01 | 40 | 26.2 | N/A | 153 | No | M | N/A |
| Illumina | 6929742057_R05C01 | 85 | 24.7 | N/A | 136 | No | M | N/A |
| Illumina | 6929742028_R06C01 | 25 | 23.5 | N/A | N/A | No | M | N/A |
| Illumina | 6929742028_R06C02 | 60 | 27.5 | N/A | 89.5 | No | M | N/A |
| Illumina | 6929742057_R05C02 | 82 | 29.4 | N/A | 112.5 | No | M | N/A |
| Illumina | 6929806060_R02C01 | 71 | 25.1 | N/A | 114 | No | M | N/A |
| Illumina | 6929742057_R01C01 | 20 | N/A | N/A | 161.5 | No | F | N/A |
| Illumina | 6929806060_R03C02 | 15 | 25.5 | N/A | 201 | No | M | N/A |
| Illumina | 6929806060_R04C01 | 28 | 24.8 | N/A | 87 | No | M | N/A |
| Illumina | 6929806060_R06C01 | 53 | 29.4 | N/A | 206 | No | M | N/A |
| Illumina | 7471147042_R04C01 | 58 | 26.1 | N/A | 115.5 | No | M | N/A |
| Illumina | 6929806060_R01C02 | 40 | N/A | N/A | 190 | No | M | N/A |
| Illumina | 6929806060_R02C02 | 60 | 26.1 | N/A | N/A | No | M | N/A |
| Illumina | 6929742057_R04C01 | 42 | 24.2 | N/A | 123 | No | M | N/A |
| Illumina | 6929742057_R01C02 | 61 | 22.9 | N/A | 156 | No | M | N/A |
| Illumina | 6929742057_R03C01 | 61 | 28.7 | N/A | 127.5 | No | M | N/A |
| Illumina | 6929742057_R02C01 | 67 | 20.8 | N/A | 285 | No | M | N/A |
| Illumina | 6929806060_R06C02 | 78 | 26.2 | N/A | 151 | No | M | N/A |
| Illumina | 6929742057_R02C02 | 36 | 24.7 | N/A | 190.5 | No | M | N/A |
| Illumina | 6929793129_R02C02 | 52 | 25.5 | N/A | 121.5 | No | F | N/A |
| Illumina | 7471147018_R04C02 | 75 | 22 | N/A | 115 | No | M | N/A |
| Illumina | 7471147018_R05C01 | 73 | 22 | N/A | 116 | No | M | N/A |
| Illumina | 7471147018_R05C02 | 77 | 24 | N/A | 168.5 | No | F | N/A |
| Illumina | 7471147018_R06C01 | 63 | 28 | N/A | 130 | No | F | N/A |
| Illumina | 7471147046_R04C02 | 38 | 22.5 | N/A | 99 | No | M | N/A |
| Illumina | 6929742038_R01C02 | 79 | 27.5 | N/A | 173 | No | F | N/A |
| Illumina | 6929742038_R02C01 | 78 | 31.3 | N/A | 151.5 | No | F | N/A |
| Illumina | 6929742038_R03C01 | 63 | 28.9 | N/A | N/A | No | F | N/A |
| Illumina | 7471147046_R05C01 | 53 | 20.2 | N/A | 169.5 | No | F | N/A |
| Illumina | 7471147046_R06C01 | 71 | 26.9 | N/A | N/A | No | F | N/A |
| Illumina | 7471147046_R06C02 | 46 | 25 | N/A | 135 | No | F | N/A |
| Illumina | 7471147042_R01C02 | 67 | 23.9 | N/A | 147 | No | F | N/A |
|  |  |  |  |  |  |  |  |  |
|  |  |  |  |  |  |  |  |  |
| Sample | **Identifier** | **Age** | **BMI** | **HbA1c** | **Glycemia** | **History of Diabetes** | **Sex** | **Diabetes Duration** |
| Illumina | 7471147042_R02C01 | 43 | 23.9 | N/A | 95 | No | F | N/A |
| Illumina | 7471147042_R03C01 | 83 | 18 | N/A | 127.5 | No | M | N/A |
| Illumina | 6929742038_R02C02 | 47 | 29.3 | N/A | 147.5 | No | F | N/A |
| Illumina | 6929742038_R04C02 | 45 | 32.9 | N/A | 131.5 | No | F | N/A |
| Illumina | 7471147042_R03C02 | 24 | 22.9 | N/A | N/A | No | M | N/A |
| Illumina | 7471147042_R04C02 | 44 | 27.7 | N/A | 114 | No | M | N/A |
| Illumina | 7471147042_R05C01 | 40 | N/A | N/A | 103 | No | F | N/A |
| Illumina | 7471147042_R06C01 | 46 | 23.9 | N/A | 111 | No | F | N/A |
| Illumina | 7471147042_R06C02 | 6 | 14.4 | N/A | N/A | No | F | N/A |
| DNA Seq | SAMN08774197 | 52 | 33.3 | 4.6 | N/A | No | M | N/A |
| DNA Seq | SAMN08784511 | 42 | 36.8 | 5.3 | N/A | No | M | N/A |
| DNA Seq | SAMN08930572 | 59 | 20.4 | 5.5 | N/A | No | M | N/A |
| dPCR | SAMN08611141 | 48 | 32.4 | 5.6 | N/A | No | M | N/A |
| dPCR | SAMN08769025 | 64 | 34.5 | 5.8 | N/A | No | M | N/A |
| dPCR | SAMN08611141 | 48 | 32.4 | 5.6 | N/A | No | M | N/A |
| dPCR | SAMN08769025 | 64 | 34.5 | 5.8 | N/A | No | M | N/A |
| dPCR | SAMN08616281 | 45 | 27.2 | 6.5 | N/A | Yes--T2D | M | 0-5 years |
| dPCR | SAMN08768765 | 65 | 42.6 | 5.9 | N/A | Yes--T2D | F | 0-5 years |
| dPCR | SAMN08769026 | 37 | 38.1 | 8.2 | N/A | Yes--T2D | F | 0-5 years |

**Supplemental Table S2: Human tissues/cell lines used as controls for Illumina 450-Array**

| GSM ID | Description |
| --- | --- |
| GSM999335 | SKMC_Methyl450K |
| GSM999336 | HIPEpiC_Methyl450K |
| GSM999337 | HeLa-S3_Methyl450K |
| GSM999338 | HepG2_Methyl450K |
| GSM999339 | Hepatocytes_Methyl450K |
| GSM999341 | K562_Methyl450K |
| GSM999343 | RPTEC_Methyl450K |
| GSM999345 | AG04450_Methyl450K |
| GSM999346 | SAEC_Methyl450K |
| GSM999347 | AG09309_Methyl450K |
| GSM999349 | AG10803_Methyl450K |
| GSM999353 | GM06990_Methyl450K |
| GSM999356 | AoSMC_Methyl450K |
| GSM999358 | NHBE_Methyl450K |
| GSM999361 | HRE_Methyl450K |
| GSM999367 | Jurkat_Methyl450K |
| GSM999374 | GM12892_Methyl450K |
| GSM999375 | GM12891_Methyl450K |
| GSM999376 | GM12878_Methyl450K |
| GSM999378 | HEEpiC_Methyl450K |
| GSM999381 | HCM_Methyl450K |
| GSM999382 | HCF_Methyl450K |
| GSM999384 | HNPCEpiC_Methyl450K |
| GSM999385 | HMEC_Methyl450K |
| GSM999390 | HRCEpiC_Methyl450K |
| GSM999391 | HPAEpiC_Methyl450K |
| GSM999395 | MethylArray_PANC-1_Methyl450K |

**Supplemental Table S3: Primers for sequencing**

| Illumina ID | RefGene Name | Forward Primer (5’ – 3’) | Reverse Primer (5’ – 3’) |
| --- | --- | --- | --- |
| cg04810745 | Intergenic | TGTTGAGTTTAGAAGTTAAGTTGGA | ACAATACCCCTAAAATACAAAA |
| cg03478610 | *PPP2R3A* | AGAGGTGGTAATTTAGGTTTGTGT | ACCATCACAATTTACTCATCCTCA |
| cg08913523 | Intergenic | GGTTTTGTGGGTTGGAAGTTAG | ACCACCCCCTCCTTCAACTA |
| cg06712013 | *SPATS2* | ATGGTTGGAGTAGATGAGAT | ACACCACTACACTCCACCCT |
| cg02482497 | *CHTOP* | TGTTGTGAGTTTTGAAGGTGTT | ACCCATTCTCTCACCTACTT |

**Supplemental Table S4: Table of all differentially methylated CpGs.** The table contains beta values for human islets and control tissues/cells, the delta-beta values between human islets and control tissues, as well as associated p-values (computed using Mann–Whitney U test followed by FDR multitesting correction). Genomic annotation of CpGs is also provided. (see Methods for more detail about the processing, and Suppl. Tables S1 and S2 for identification of human islets and non-islet tissues from the Illumina IDs).

**Supplemental Figure S1: Experimental Workflow.**

**Supplemental Figure S2: Immunofluorescence staining of flow-sorted human β cells.** Human islets were sorted by flow cytometry after Newport Green staining. Sorted α-cells (top left quadrant) and β-cells (bottom right quadrant) were immunostained for glucagon or insulin, respectively, and DAPI.
